# CCR2 improves tumor directed CAR-T cell trafficking in ovarian cancer

**DOI:** 10.3389/fphar.2025.1651526

**Published:** 2025-12-05

**Authors:** Raj Kumar, Irva E. Veillard, Mengyao Xu, Linah Al-Alem, Bo R. Rueda, Oladapo O. Yeku

**Affiliations:** 1 Massachusetts General Hospital Cancer Center, Boston, MA, United States; 2 Meigs Division of Gynecologic Oncology, Vincent Department of Obstetrics and Gynecology, Massachusetts General Hospital, Boston, MA, United States; 3 Massachusetts General Hospital Cancer Center, Harvard Medical School, Boston, MA, United States

**Keywords:** ovarian cancer, MUC16, chimeric antigen receptor T-cells, CAR-T cells, armed CAR-T cells, chemokines

## Abstract

Solid tumors present a significant challenge to Chimeric Antigen Receptor (CAR) -T cell therapy, primarily due to limited T-cell infiltration and persistence in the tumor microenvironment. Cancers with predominantly peritoneal metastasis like ovarian cancer pose a substantial trafficking challenge to systemically administered CAR-T cell therapy. To identify chemokines that may guide CAR-T cells to tumor sites, we evaluated chemokine expression in primary and metastatic tumor samples from patients with ovarian cancer by immunohistochemistry. After identifying CCL2 as the most common chemokine expressed in both primary and metastatic disease, we validated CCL2 levels in serum samples from these patients. We found that CCL2 and CCL4 were commonly expressed in several ovarian cancer cell lines, a patient-derived tumor cell line and patient serum samples *in vitro*. We engineered armed Muc16/CA-125-directed CAR-T cells with cognate receptors for CCL2 and CCL4 and showed significant *in vitro* and *in vivo* tumor-directed trafficking. Finally, intravenously administered CCL2-armored CAR-T cells significantly prolonged survival in peritoneal tumor bearing mice. In conclusion, we identified CCL2 as a commonly expressed chemokine in patients with local and metastatic ovarian cancer and successfully demonstrate that armed CAR-T cells with enhanced homing to CCL2-expressing ovarian cancer are more efficacious due to improved peritoneal trafficking *in vivo*.

## Introduction

Despite favorable outcomes in hematologic malignancies, Chimeric Antigen Receptor (CAR) T-cell therapy has yet to show any clinical benefits for the management of solid tumors (Zhang et al., 2016). In epithelial ovarian cancer (EOC), several promising preclinical CAR constructs directed against Folate Binding Protein, MUC-1, mesothelin and Lewis-Y antigens have been reported (Carpenito et al., 2009; Kershaw et al., 2002; Parker et al., 2000; Wang et al., 1998; Westwood et al., 2005; Wilkie et al., 2008), yet none of these have led to any significant clinical benefits. Furthermore, CAR T-cells directed against the alpha-folate receptor overexpressed on ovarian cancer cells showed limited *in vivo* persistence and did not lead to any meaningful antitumor responses in patients with relapsed ovarian cancer despite encouraging preclinical results (Kershaw et al., 2006). A prevailing hypothesis for the lack of efficacy in solid tumors is the presence of an immunosuppressive tumor microenvironment. For instance, regulatory T-cells (Treg) (Curiel et al., 2004; Wolf et al., 2005), tumor associated macrophages (TAMs) (Duraiswamy et al., 2013a; Duraiswamy et al., 2013b; Quatromoni and Eruslanov, 2012) and myeloid derived suppressor cells (MDSCs) (Bak et al., 2008) have all been reported to mediate T-cell suppression. A less studied factor has been the relatively poor trafficking of CAR T-cells to the tumor. Our group and others have made improvements to the CAR design to overcome the hostile immunosuppressive tumor microenvironment (Yeku and Brentjens, 2016), however, none of these interventions have been shown to improve CAR-T cell trafficking to solid tumor lesions. Even modifications that improve CAR-T cell potency such as co-expression of IL-12 did not necessarily improve trafficking in EOC and the resulting IL-12 armored CAR-T trial involved intraperitoneal and intravenous administration of these engineered CAR-T cells (Koneru et al., 2015). Other forms of armored CAR- T cells such as the recently published epithelial growth factor-targeting CAR-T cells which were armored to also secrete EGFR-targeting bispecific T-cell engagers required intracavitary delivery (Choi et al., 2024).

Notably, there is no shortage of monocyte and macrophage infiltration of metastatic peritoneal disease in EOC (Wang et al., 2006) and EOC has been shown to secrete several chemokines such as CXCL10, CXCL12, and CXCL16 (Zsiros et al., 2015). We hypothesized that decreased CAR-T cell trafficking in EOC could be due to chemokine/chemokine receptor mismatch. In other disease models such as Hodkin lymphoma, neuroblastoma, and mesothelioma, chemokine receptor modified CAR-T cells have been shown to improve tumor-directed trafficking and cytotoxicity (Craddock et al., 2010; Di Stasi et al., 2009; Moon et al., 2011).

In this report, we evaluated EOC tissue samples from primary and metastatic sites to determine which chemokines were more likely to be concordantly expressed. After validating that these putative chemokine candidates could also be measured in patients’ serum samples, we identified chemokine candidates that were secreted by cultured patient tumor cells and ovarian cancer cell lines. Cognate chemokine receptors were subsequently co-expressed in second generation CD28-costimulated CAR-T cells targeting the retained portion of the tumor associated antigen Muc16/CA125 (4H11) and evaluated for tumor-directed trafficking *in vitro* and *in vivo*. We show that CCR2-armed CAR-T cells (4H11-CCR2) exhibit improved trafficking *in vitro*, improved trafficking and disease control *in vivo* and increased survival in peritoneal EOC tumor-bearing mice.

## Materials and methods

### Immunohistochemistry

Paraffin blocks representing surgical cases from patients with a diagnosis of high grade serous ovarian cancer were retrieved from the Vincent Center for Reproductive Biology MGH Gyn Repository. Samples represent patients with treatment-naive disease or interval cytoreduction after neoadjuvant chemotherapy. Matched samples representing, primary ovarian cancer, adnexal tumor, omental tumor, large bowel metastasis and liver metastasis where available. In total, twelve unique patients were identified (MGH-1-12), with all except MGH-7 having more than one site of disease available for analysis. Matched banked serum samples were available for MGH-1, MGH-3, MGH-5, MGH-7, and MGH-11. All samples were obtained under secondary use protocol #2020P002142. Paraffin blocks representing tumor samples from each patient were serially sectioned and probed with Anti-IP10 (CXCL10) (abcam9807), Anti-CCL2 (Sigma HPA019163), Anti-CCL28 (R&D MAB7171-sp) or Anti-SDF-1 (CXCL12) according to manufacturer’s dilution instructions. Isotypes Anti-Rabbit (DA1E) mAb IgG XP® Isotype Control (Cell Signaling Technology 3900S) and Anti-Mouse IgG1 control isotype (Dako/Agilent X093101-2) were used as controls according to manufacturer’s instructions. Staining has been described previously (Al-Alem et al., 2024). Briefly, antigen retrieval was performed using 10 nM sodium citrate solution at 120 °C using a pressure cooker for 15 min. Tissues were incubated with 3% H_2_O_2_ (Cat #S25359, Thermo Fisher) for 20 min, then blocked with 6% serum cocktail (normal Horse Cat #S2000, Bovine Cat # SP5050, Goat Cat #S1000 serum from Vector Labs, Burlingame, CA) for 20 min. Tissues were then incubated with indicated primary antibodies or isotype control, or no primary antibody diluted in the blocking cocktail. After washing with PBST, slides were incubated for 45 min with anti-mouse antibody (Santa Cruz, Dallas, TX). To visualize the staining, 3,3′-diaminobenzidine (DAB) was used (Cat # SK-4100; Vector stains, Burlingame, CA). Slides were counterstained with hematoxylin (Cat # CS402-1D Thermo Fisher Scientific) and Scott’s water. Finally, slides were dehydrated and mounted with coverslips. Intracellular positivity was scored by the intensity of the staining. A score of 0 meant minimal staining in less than 5% tumor cells, or no staining was identified. A score of 1+ indicated minimal to moderate staining in 5%–24% of tumor cells. A score of 2+ indicated moderate to strong staining in 25–50% of tumor cells.

### Chemokine quantification

Chemokine levels were measured in ovarian cancer cell lines and clinical serum samples using a quantitative multiplex chemokine assay. Supernatants were collected from cell cultures after 48 h of incubation, and serum samples were thawed on ice. The levels of key chemokines, including MCP-1 (CCL2), MIP-1β (CCL4), RANTES (CCL5), GROα (CXCL1), SDF-1α (CXCL12), and CXCL8 (IL-8), were quantified using a Luminex-based multiplex bead assay kit (ProcartaPlex™ Human Cytokine & Chemokine (Thermofisher scientific) according to the manufacturer’s instructions. All samples were run as technical triplicates. For each individual patient sample and healthy donor serum sample, the mean value from technical replicates are presented. Data were analyzed using a Luminex xMAP system.

### Cell lines and culture conditions

The following ovarian cancer cell lines were utilized for this study: OVCAR3, OVCAR4, OVCAR8, CAVO362, A2780, SKOV3 (Muc16 negative), and SKOV3Muc16 + (Muc16+) and ET2 (high grade serous ovarian cancer cells grown from ascites of patient with platinum resistant ovarian cancer). These cell lines were cultured in RPMI-1640 medium supplemented with 10% fetal bovine serum (FBS), 1% penicillin-streptomycin, and 1% L-glutamine (Thermo Fisher Scientific). All cultures were maintained at 37 °C in a humidified atmosphere containing 5% CO_2_. Cells were passaged every 2–3 days to maintain healthy logarithmic growth. Cells were routinely tested for *mycoplasma* contamination.

### Molecular cloning of chemokine receptors

To generate armed Muc16 CART cells with chemokine receptors, CCR2 and CCR5 chemokine receptors were cloned into a second-generation Muc16 CAR retroviral vector. Cloning was performed using standard molecular cloning techniques. The 4H11-H1L2 Muc16CAR ligated at *Xho*I/*EcoR*I restriction sites (Forward 5′-GGC​CCA​CCA​TGG​CTC​TCC​C and Reverse 5′-TAG​CGA​GGG​GGC​AGG​GC), CCR2b ligated at *BamH*I/*Sal*I (Forward 5′-ATG​CTG​TCC​ACA​TCT​CGT​TC and Reverse 5′-TTA​TAA​ACC​AGC​CGA​GAC​TTC​C) and CCR5, ligated at *BamH*I/*Sal*I (Forward 5′-ATG​GAT​TAT​CAA​GTG​TCA​AGT​C and Reverse 5′-TTA​CAA​GCC​CAC​AGA​TAT​TTC). Briefly, CCR2b and CCR5 were amplified by polymerase chain reaction (PCR) from source vector plenti-CCR2b-puro and plenti-CCR5-puro and inserted downstream to Muc16 CAR separated by P2A element into pMSCV-puro vector (Clontech) using restriction enzymes. The plasmid constructs pMSCV-4H11-H1L2 Muc16-P2A-CCR2b, pMSCV-4H11-H1L2 Muc16-P2A-CCR5 and additional control vectors, pMSCV-CCR2b, pMSCV-CCR5, and pMSCV-CCR5-P2A-CCR2b, were verified by restriction digestion followed by Sanger sequencing.

### Retroviral packaging and production

Retroviral vector particles for the armed CCR-Muc16 CART constructs were generated using Phoenix amphotropic packaging cells. Per the manufacturer’s instructions, transfection was performed using lipofectamine 3000 (Invitrogen). Viral supernatants were collected 48- and 72-h post-transfection, filtered through an SFCA membrane 0.45 μm filter (Corning, River St, Oneonta NY), and stored at −80 °C for further use.

### Transduction of human T Cells

CAR-T cell transduction has been reported previously (Yeku et al., 2017). Human peripheral blood mononuclear cells (PBMCs) were isolated from healthy donors using Cell Preparation Tube (CPT) Ficoll-based density gradient centrifugation. Briefly, cells were centrifuged at 1,700 g for 20 min followed by isolation of the buffy coat. Trypan blue dye was used to stain dead cells and determine the number of viable cells per milliliter using a BioRad cell counter device. PBMCs were activated using PHA 2 μg/mL (R&D Minneapolis MN) and IL-2, 100 IU/mL (R&D Minneapolis MN). After 48 h of activation, T cells were transduced with the Muc16 CAR-T retroviral supernatants containing either the CCR2 or CCR5 chemokine receptors. Activated T cells were seeded at 3 million cells per well of a six-well plate coated with 20 ug/mL recombinant human fibronectin fragment (RetroNectin, (TaKaRa Bio Inc) layover with 3 mL 0.45 micron (SFCA corning) filtered armed-Muc16 CART retrovirus supernatant and spinoculated at 1,800 g, 30 °C for 1 hour every day for three consecutive days. On day six, transduction efficacy was determined using flow cytometry to detect Muc16 CAR (anti-F(ab)_2_-FITC antibody) and anti-CCR2-APC antibody for chemokine receptor expression and CD3 by anti-CD3-PE-Cy7 antibody. Fluorescence compensation was performed according to manufacturers instructions.

### *In Vitro* chemotaxis assays

To assess CAR-T cell chemotactic response using a transwell assay, indicated cells were harvested and resuspended in 0.5%FBS RPMI+1xP/S medium at 1.6 × 10^5^ cells/mL. In the bottom well, 500 µL medium with the chemoattractant (CCL2 or CCL4 at 0.01 ug/mL) was added, and 0.5% FBS RPMI+1x P/S medium was added to the control wells. 300 μL of cell suspension (50,000 cells) to the insert (Corning, 8 μm pore size) and placed in the well followed by incubation for 12 h. Following this, 2 uM Calcein AM (2 uL/mL of 1 mM) was added to enumerate cells in the lower chamber via fluorescence using a spectrophotometer SpectraMax iD3 plate reader (Molecular Devices Old Connecticut Path, Framingham, MA). Additionally, live cell imaging was conducted using an IncuCyte system (Sartorius) to track T cells’ real-time migration and chemotaxis toward chemokines. T cells were labeled with Calcein AM 2 µM and migration was monitored using automated imaging every 4 h for 36 h. Migration of cells from the upper chamber to the lower chamber was quantified based on fluorescent image data, i.e., the number of cells that remained on the Clearview insert and normalized to day one count.

### Cytotoxicity assay

SKOV3-Muc16+ and OVCAR3 cells were seeded at a density of 20,000 cells per well in 96-well plates and allowed to adhere overnight in a 37 °C, 5% CO_2_ incubator. Effector T-cells were added at effector-to-target (E:T) ratios of 1:1 and 5:1. After 48-h, an LDH release cytotoxicity assay was employed to assess the extent of cell lysis as per manufacturer guidelines. Specific cytolysis was calculated using the formula; % specific lysis = 100 × (sample lysis–spontaneous lysis)/(maximal lysis–spontaneous lysis). For real-time cytotoxicity assessment, an IncuCyte live-cell imager was used, and cytotoxicity was evaluated over 120 h. Point estimates at 48 h, 72 h and 116 h are also shown.

### Animal studies, *in vivo* trafficking

Female NSG mice aged 8–12 weeks were injected with OVCAR3 cell at 5 × 10^6^ cells per mouse intraperitoneally. On day 21 post-cancer cell inoculation, mock transduced T-cells, unarmed second-generation MUC16-CAR T-cells (4H11) or armed MUC16-CAR T cells (4H11-CCR2b, 4H11-CCR5) were administered intravenously via tail vein injection at a dose of 2 × 10^6^ cells per mouse. For evaluation of *in vivo* trafficking at the indicated timepoints, animals were euthanized and subjected to peritoneal washes and splenectomy. Flow cytometry was used to enumerate peritoneal tumor cells and CAR-T cells. Three mice per group were used for each *in vivo* trafficking timepoint. Ten mice per group were used for survival evaluation. Tumor progression was monitored twice weekly by assessing abdominal distension.

### Statistical analysis

Kaplan-Meier survival curves were used to assess the survival of mice across different treatment groups. and other analysis were performed using unpaired two-tailed T-test (p value <0.05 considered as significant). All calculations were performed using Prism 7 (GraphPad) software.

### Study approval

All murine studies were done in accordance with the Massachusetts General Hospital Institutional Animal Care and Use Committee approved protocol 2020N000045. Excess Human Material secondary use protocol 2020P002142. All experiments were performed in accordance with relevant guidelines and regulations.

### Data availability

All data generated and analyzed during this study are included in the published article and Supplementary Material. Mouse models and antibodies used are commercially available. Cell lines available upon request.

## Results

### Tumor chemokine analysis

**FIGURE 1:**
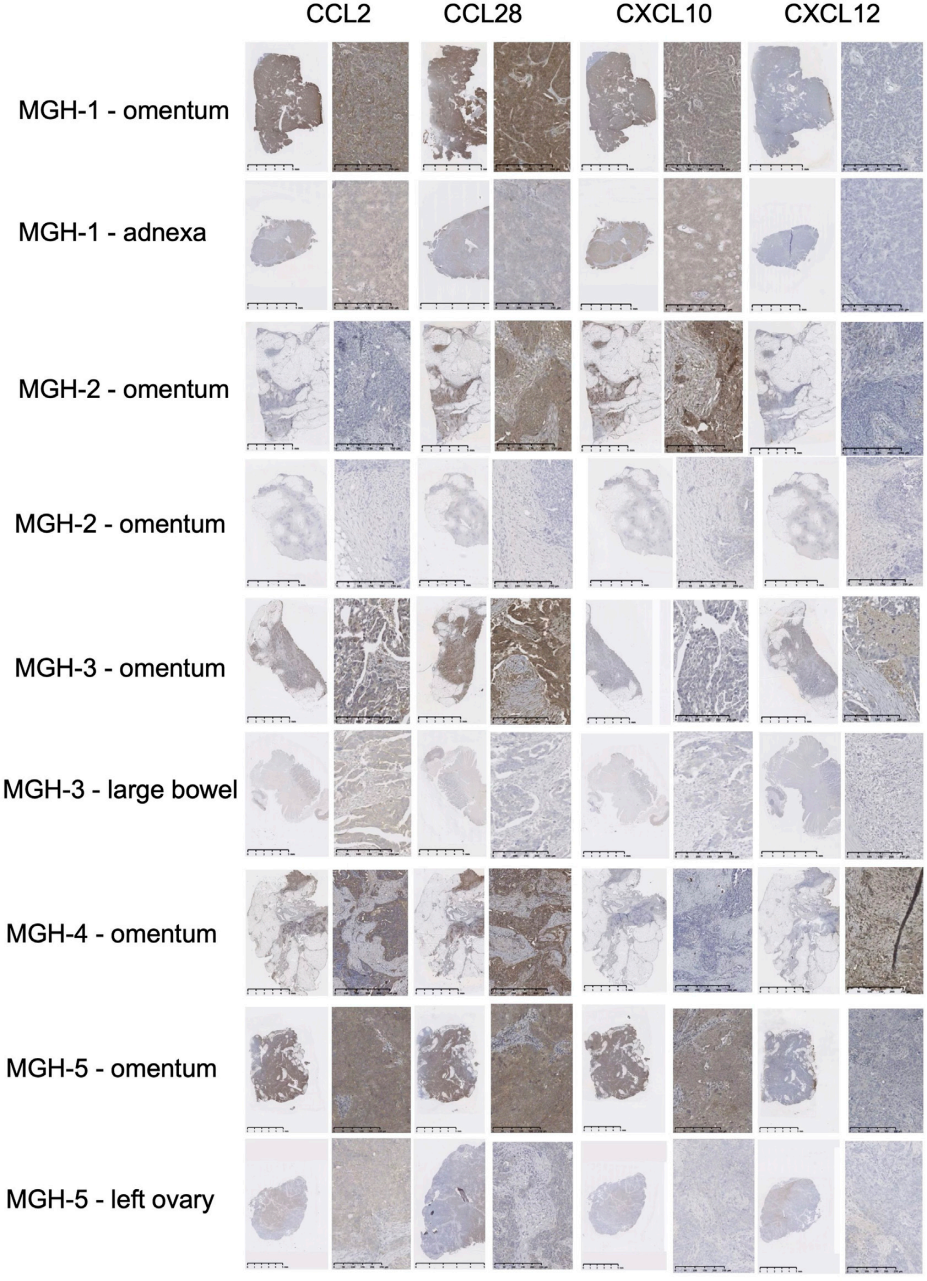
Chemokine immunohistochemistry staining of ovarian cancer tissue specimens. Expression levels of CCL2, CCL28, CXCL10 and CXCL12 were evaluated. Samples from patients MGH-1 – MGH-5 are shown.

Ovarian cancer chemokine landscape profiling revealed chemokines such as CCL2, CCL4, CCL5, CXCL10, and CXCL12 as potential candidates in ovarian cancer (Zsiros et al., 2015). We selected CCL2, CCL28, CXCL10 and CXCL12 as potential candidates for further validation due to their roles as monocyte chemoattractants. We evaluated matched patient samples representative of omental sites, ovary, adnexa, large bowel, and liver metastasis and preformed immunohistochemistry (IHC) with antibodies against CCL2, CCL28, CXCL10 and CXCL12 (Figure 1). Representative IHC images are shown in Figure 1, and a tabulation of the full results are shown in Supplementary Table S1 and Supplementary Figure S1. Samples from twelve patients were analyzed and we found concordant expression of CCL2 in all but one patient, MGH-8. We also found concordant CCL28 in all but 3 of our paired samples; MGH-1, MGH-2 and MGH-3. CXCL-10 and CXCL-12 showed varying degrees of expression in various tumor sites but did not show as much correlation between sites as CCL2 and CCL28.

### Patient, healthy donor, and cell line chemokine evaluation

**FIGURE 2:**
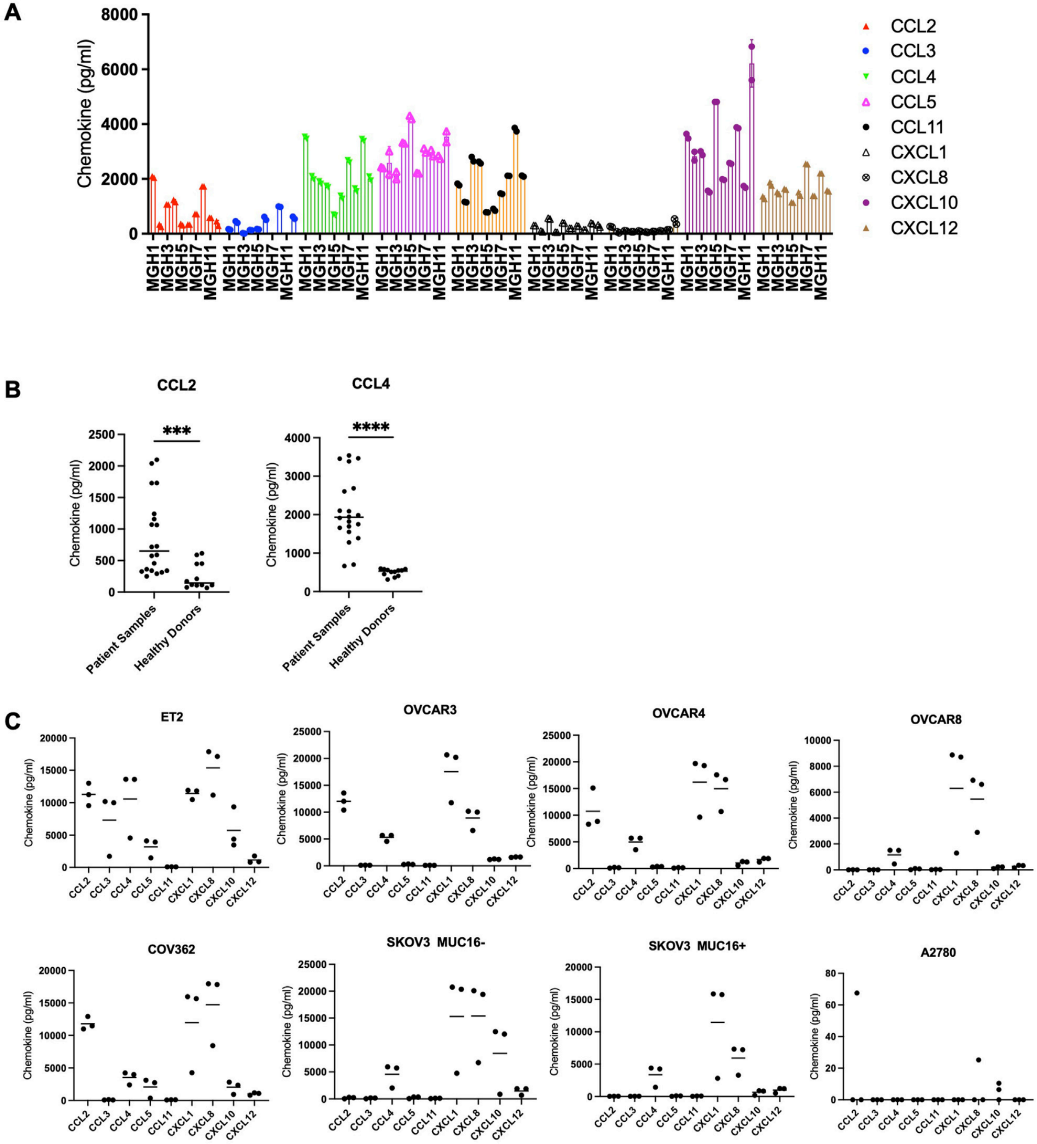
**(A)** Multiplexed chemokine analysis on serum samples from patients MGH1, MGH3, MGH5, MGH7, and MGH11. **(B)** Chemokine levels of CCL2 and CCL4 from healthy donors and patients with ovarian cancer are shown. ***; p = 0.0004, ****; p < 0.0001. **(C)** Multiplexed chemokine analysis on ovarian cancer cell lines and patient-derived cell line ET2. Expression levels of CCL2, CCL3, CCL4, CCL5, CCL11, CXCL11, CXCL8, CXCL10, and CXCL12 are shown. Data shown are representative of three independent experiments.

Next, we evaluated patient serum samples for chemokine secretion. From the subset of twelve patient samples used for IHC analysis, only five had serum samples (MGH-1, MGH-3, MGH-5, MGH-7, MGH-11). Enzyme-Linked Immunosorbent Assay (ELISA) analysis revealed measurable levels of CCL-2, CCL-4, CCL-5, CCL-11, CXCL-10 and CXCL-12 (Figure 2A). Using female healthy donors with no history of ovarian cancer, we were also able to detect levels of CCL2, CCL4, CCL5, CXCL10 and CXCL12 (Supplementary Figure S2). However, levels of CCL2 and CCL4 were significantly increased in patients with ovarian cancer compared to healthy donors (Figure 2B). In a cultured cell line derived from the ascites of a patient with platinum resistant ovarian cancer (ET2), elevated levels of CCL2, CCL3, CCL4, CCL5, CXCL1, CXCL8 and CXCL10 were detected in the cultured media (Figure 2C). In a panel of 6 cell lines including an isogenic SKOV3 cells with or without Muc16 expression, we found varying degrees of chemokine expression with CCL2, CCL4, CXCL1, and CXCL8 being the predominant chemokines (Figure 2C). Based on the patient IHC and serum samples, and cell lines, we nominated CCL2 and CCL4 as leading chemokine candidates and their cognate receptors, CCR2b and CCR5, were selected for co-expression in CAR-T cells.

### Chemokine receptor armored CAR-T cells and *in vitro* chemotaxis

**FIGURE 3:**
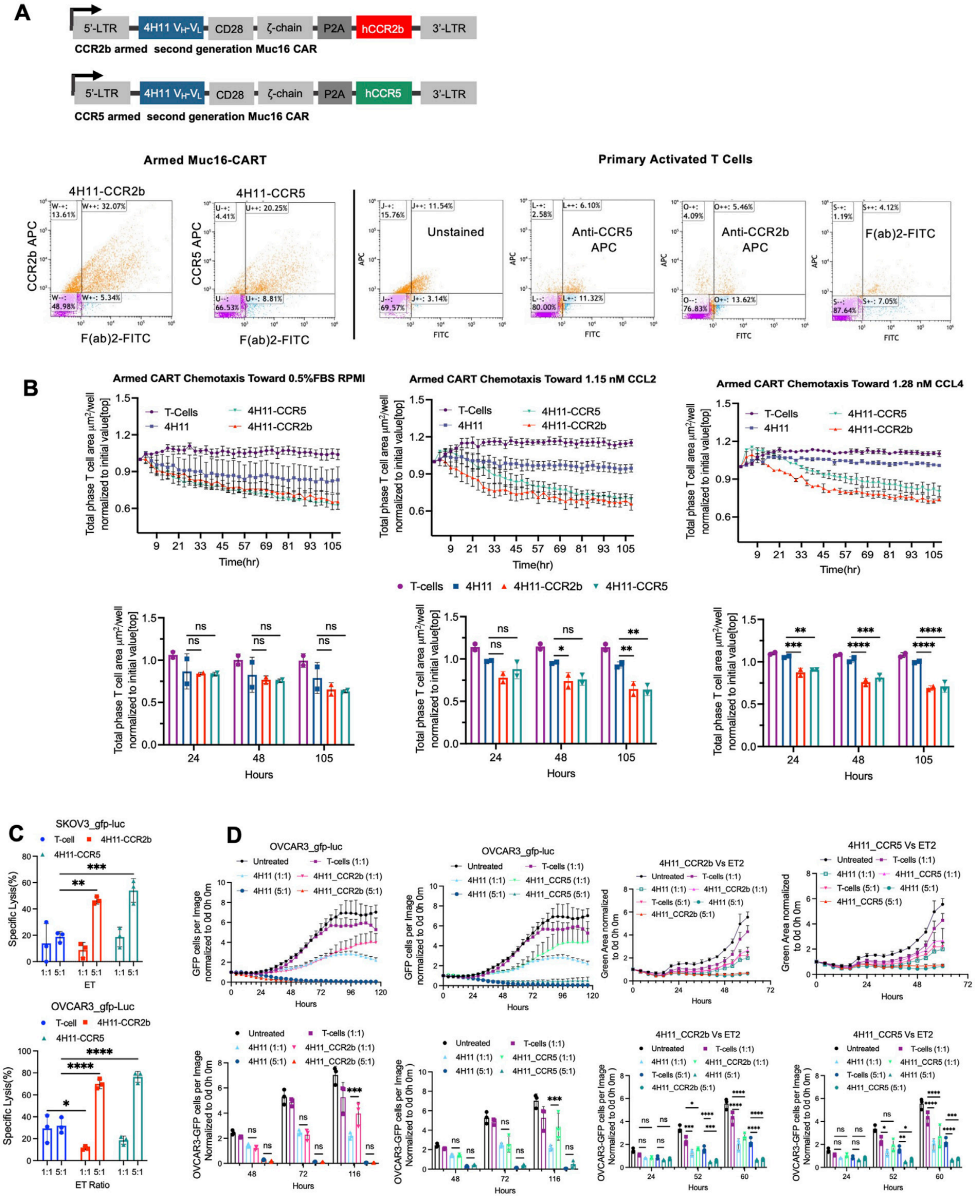
**(A)** Cartoon representation of CCR2b and CCR5 armed CARs. Flow cytometry confirmation of anti-Muc16^ecto^ scFv (F(ab)2-FITC) and CCR2b (APC) or CCR5 (APC) CAR-T cells. **(B)** *In vitro* chemotaxis of untransduced T-cells, second-generation anti-Muc16^ecto^ CAR-T cells, CC2b armed anti-Muc16^ecto^ CAR-T cells (4H11-CCR2b), or CCR5 armed anti-Muc16^ecto^ CAR-T cells (4H11-CCR5) (top chamber) in response to FBS, CCL2 or CCL4 (bottom chamber). CAR/T-cells were scored and counted on the top chamber. Decreasing signal indicates migration to the bottom chamber towards the chemoattractant. Pooled quantification of chemotaxis from two independent biological replicates at 24 h, 48hrs., and 108 h are shown below the graphs. ns: not significant. *; p < 0.05. **; p < 0.004. ****; p < 0.0001. **(C)** *In vitro* cytotoxicity of 4H11-CCR2b or 4H11-CCR5 armed CAR-T cells against SKOV3 (top panel) or OVCAR3 (bottom panel) cells at Effector to Target (E:T) ratio of 1:1 and 5:1 are shown. Figures are representative of two independent replicates. **(D)** Panel 3D should read: **(D)**. *In vitro* evaluation of second 4H11 and 4H11-CCR2b or 4H11 and 4H11-CCR5 armed CAR-T cells against OVCAR3 cells and patient derived organoid ET2 at E:T of 1:1 and 5:1 over 120 hrs. Pooled quantification of cytotoxicity from two independent biological replicates at 48 hrs., 72 hrs., and 116 hrs are shown. ****; p < 0.0001.

We have previously published a second generation CD28ζ co-stimulated CAR-T cell targeting the tumor retained portion of Muc16/CA-125 (Lee et al., 2024) (4H11) and generated armored versions expressing CCR2b (4H11-CCR2) and CCR5 (4H11-CCR5) (Figure 3A). We validated co-expression of the Muc16-targeting scFv and CCR2b or CCR5 using flow cytometry (Figure 3A). Second generation 4H11 CAR-T cells did not express CCR2b or CCR5, 4H11-CCR2 CAR-T cells did not express CCR5, and 4H11-CCR5 CAR-T cells did not express CCR2b (Supplementary Figure S3 **)**. To evaluate the chemotactic potential of these cells, we used a transwell migration assay with either 4H11, 4H11-CCR2b or 4H11-CCR5 on the top well and either FBS, CCL2 or CCL4 in the bottom chamber (Figure 3B). All three CAR-T cells migrated towards the bottom chamber containing FBS but there was no difference among them until 108 h where 4H11-CCR2b and 4H11-CCR5 showed modest increases in migration over 4H11. In contrast, CCL2 led to an increase in 4H11-CCR2b migration at 48 h and 108 h 4H11-CCR5 also showed improvement in migration in response CCL2. Similarly, CCL4 also led to an increase in 4H11-CCR2b and 4H11-CCR5 migration over 4H11.

To ensure that 4H11-CCR2b and 4H11-CCR5 retained cytotoxicity, we cocultured both armored CAR-T cells with either OVCAR3 or SKOV3-Muc16+ ovarian cancer tumor cells for 48 h at effector to target (E:T) ratios of 1:1 and 5:1 (Figure 3C). Both CAR-T cells demonstrated cytotoxicity against ovarian cancer cells *in vitro*. Next, we evaluated 4H11-CCR2b and 4H11-CCR5 cytotoxicity against OVCAR3 and patient derived tumor organoid ET2 over 120 h and found sustained cytotoxicity over time in both the cell line and patient derived organoid (Figure 3D).

### *In vivo* chemotaxis and efficacy

**FIGURE 4:**
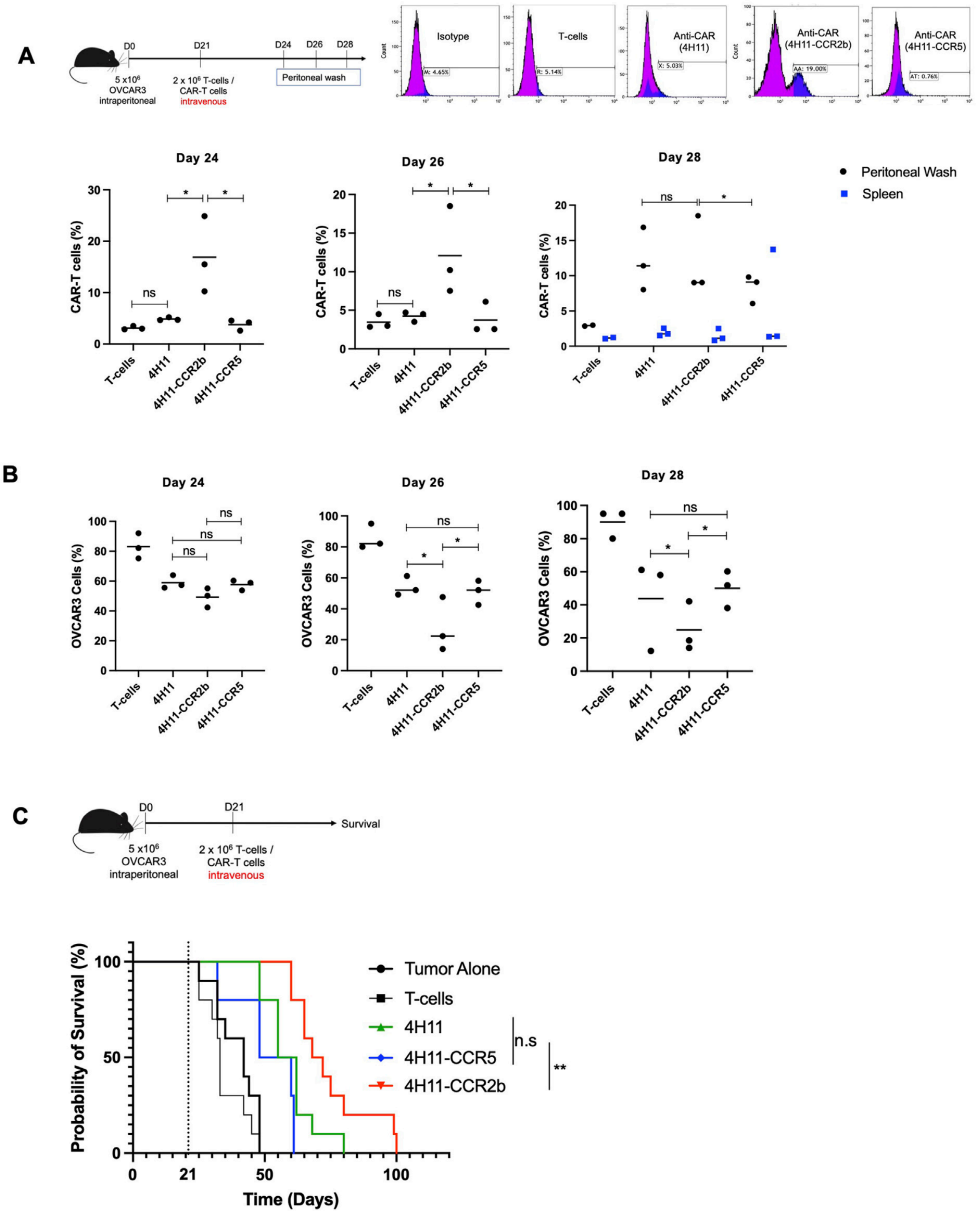
**(A)** Peritoneal washes were performed on tumor bearing mice treated intravenously with untransduced T-cells, 4H11, 4H11-CCR2b or 4H11-CCR5 CAR-T cells on day 24, day 26, and day 28 after tumor inoculation (day 3, day 5, day 7 after treatment) and evaluated for peritoneal CAR-T cells by flow cytometry. Data shown are pooled from three mice per group. ns: not significant. *; p < 0.05. **(B)** Peritoneal washes were performed on tumor bearing mice treated intravenously with untransduced T-cells, 4H11, 4H11-CCR2b or 4H11-CCR5 CAR-T cells on day 3, day 5, and day 7 after treatment and evaluated for peritoneal OVCAR3 cells by flow cytometry. On day 7, spleen samples were also evaluated. ns: not significant. Data shown are pooled from three mice per group *; p < 0.05. **(C)** OVCAR3 tumor-bearing mice treated with untransduced T-cells, 4H11, 4H11-CCR2b or 4H11-CCR5 CAR-T cells on day 21 after tumor inoculation and evaluated for survival. **; p < 0.05. Data shown are pooled from two independent experiments with five animals per group per experiment.

To evaluate if 4H11-CCR2b and 4H11-CCR5 migrated to peritoneal disease *in vivo*, we inoculated female NSG mice with OVCAR3 cells intraperitoneally (i.p) on day 0. On day 21, we injected mock transduced T-cells, 4H11, 4H11-CCR2b or 4H11-CCR5 intravenously. On day 24, day 26 and day 28, we performed peritoneal washes on these animals and quantified peritoneal tumor cells and CAR-T cells (Figure 4A). We also evaluated the number of CAR-T cells in the spleen on day 28 since this was one of the sites that i.v administered CAR-T cells tend to naturally home to. As shown in Figure 4A, there was significantly more 4H11-CCR2b in the peritoneum of tumor bearing mice on days 24, 26, and 28 after i.v treatment with minimal amounts in the spleen on day 28. In contrast, 4H11-CCR5 trafficking to the peritoneum was similar to 4H11 with a significant splenic sequestration on day 28. Peritoneal tumor cells were similar on day 24 among 4H11, 4H11-CCR2b and 4H11-CCR5 treated mice (Figure 4B). However, there was significantly less tumor cells in 4H11-CCR2b treatment mice on day 26 and 29 compared to 4H11 and 4H11-CCR5 treated mice. This decrease in tumor burden extended to a survival advantage in 4H11-CCR2b (median survival of 70 days) treated mice compared to 4H11 (median survival of 58.5 days) and 4H11-CCR5 (median survival of 54 days) (Figure 4C).

## Discussion

In this report, we show that ovarian cancer tumor cells elaborate several chemokines from both the primary ovarian lesion and from disease located at distant metastatic sites. We show that although there is heterogeneity in the chemokines from different disease sites, there is some overlap with chemokines such as CCL2. CCL2 is also readily detected in the serum of patients with ovarian cancer. Muc16-directed CAR-T cells engineered to express CCR2, the cognate receptor to CCL2, demonstrate better *in vitro* and *in vivo* tumor-directed trafficking and improve survival in tumor-bearing mice.

Ineffective tumor-directed trafficking of CAR-T cells in solid tumors is a known challenge and there have been several efforts to overcome this including direct intracavitary instillation of CAR-T cells (Koneru et al., 2015; Choi et al., 2024; Adusumilli et al., 2021). Furthermore, efforts to optimize CAR-T cells using overexpression of chemokine receptors have also been explored. Craddock et al. (2010) evaluated a GD2-directed CAR-T that was additionally modified to express CCR2. They found increased tumor homing and antitumor activity in a preclinical neuroblastoma model. Mesothelin-specific CAR-T cells expressing CCR2b have also been shown to traffic effectively and eradicate established mesothelioma tumor xenografts and preclinical models of lung cancer (Moon et al., 2011; Wang et al., 2021). A difference between our approach and other published approaches is the use of patient samples from primary and metastatic disease along with patient serum to identify the most suitable chemokine-chemokine receptor combination. We also show that CCR2 armored CAR-T cells improve trafficking in a disseminated peritoneal model of ovarian cancer.

Not all chemokine receptors are suitable for the purposes of improving CAR-T cell trafficking and efficacy. We were surprised to find that CCR5 did not improve *in vivo* trafficking or survival in tumor bearing mice despite detection of CCL4 in the patient serum and favorable *in vitro* results. Some studies have suggested redundant roles for CCL2 and CCL4 (Mikucki et al., 2015) so we expected that our CCR2 and CCR5 armored CAR-T cells would be equivalent in terms of *in vitro* and *in vivo* tumor-directed homing and efficacy. Therefore, it was surprising to find that CCR5-armored CAR-T cells preferentially trafficked to the spleen to the detriment of anti-tumor efficacy. A possible explanation for this finding is the secretion of CCL5 from splenic lymphocytes which has a higher affinity than CCL4 for CCR5 (Isaikina et al., 2021).

We did not see any differences in cytotoxicity between second generation 4H11 and armed 4H11-CCR2b and 4H11-CCR5 CAR-T cells. This is due to the fact that the genetic modifications we made were only designed to improve chemotaxis and do not affect *in vitro* cytotoxicity. However, the 4H11-CCR2b CAR-T cells are able to traffic to the peritoneal cancer more rapidly than 4H11 or 4H11-CCR5, and this led to increased cytotoxicity and survival *in vivo*. Our results align with the findings that efficient trafficking and time-to-antigen encounter and activation is critical for CAR T-cell function (Yeku et al., 2017; Adusumilli et al., 2014).

In conclusion, we show that CAR-T cell efficacy can be improved in ovarian cancer via co-expression of the chemokine ligand CCLR2b. The cognate chemokine for this receptor was identified via a screen on chemokine candidates enriched in both primary and metastatic disease sites.

## Data Availability

The original contributions presented in the study are included in the article/Supplementary Material, further inquiries can be directed to the corresponding author.

## Supplementary material

SUPPLEMENTARY FIGURE1 Chemokine immunohistochemistry staining of ovarian cancer tissue specimens. Expression levels of CCL2, CCL28, CXCL10 and CXCL12 were evaluated. Samples from patients MGH-6 - MGH-12 are shown..

SUPPLEMENTARY FIGURE 2 Multiplexed chemokine analysis on serum samples from six healthy donors (Donor 1- 6) are shown.

SUPPLEMENTARY FIGURE 3 Multiplexed chemokine analysis on serum samples from six healthy donors (Donor 1- 6) are shown. Flow cytometry evaluation of CCR2b and CCR5 expression on 4H11, 4H11-CCR2, and 4H11-CCR5 CAR-T cells.

SUPPLEMENTARY TABLE S1 Scoring of chemokine immunohistochemistry staining of ovarian cancer tissue specimens. Expression levels of CCL2, CCL28, CXCL10 and CXCL12 from patients MGH1 - MGH12 are shown. 0; no expression, 1+; minimal to moderate, 2+; moderate to strong staining.

## Abbreviations

APC, Allophycocyanin; CAR, Chimeric Antigen Receptor; EOC, epithelial ovarian cancer; E, T: Effector-to-target ratio; FITC, Fluorescein isothiocyanate; IL-12, Interleukin 12; Muc16, Mucin 16; MDSCs, myeloid derived suppressor cells; scFv, Short chain variable fragment; TAMs, tumor associated macrophages; Treg, regulatory T-cells.
