# Supplementary material for: CCR2 improves tumor directed CAR-T cell trafficking in ovarian cancer
Source: Front Pharmacol. 2025 Dec 5;16:1651526. doi: 10.3389/fphar.2025.1651526 (PMC12714929; doi:10.3389/fphar.2025.1651526)
Supplement: Supplementary file 1 [file DataSheet1.pdf]

Supplementary Materials for

CCR2 improves tumor directed CAR-T cell trafficking in ovarian cancer

Raj Kumar[1], Irva E. Veillard[1], Mengyao Xu[1], Linah Al-Alem[2], Bo R. Rueda[2,3], and Oladapo O. Yeku[3*]

Supplementary Figure 1

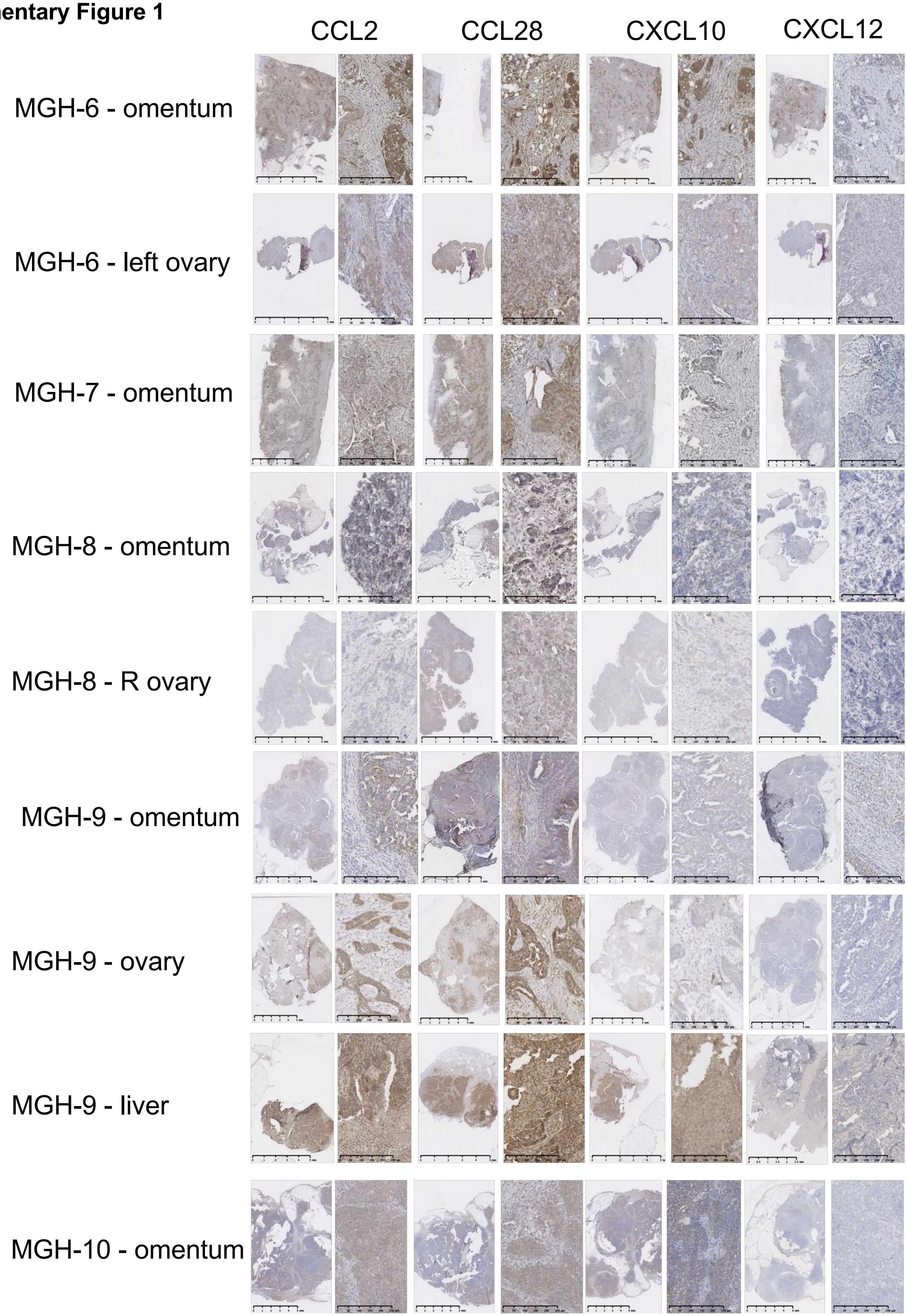

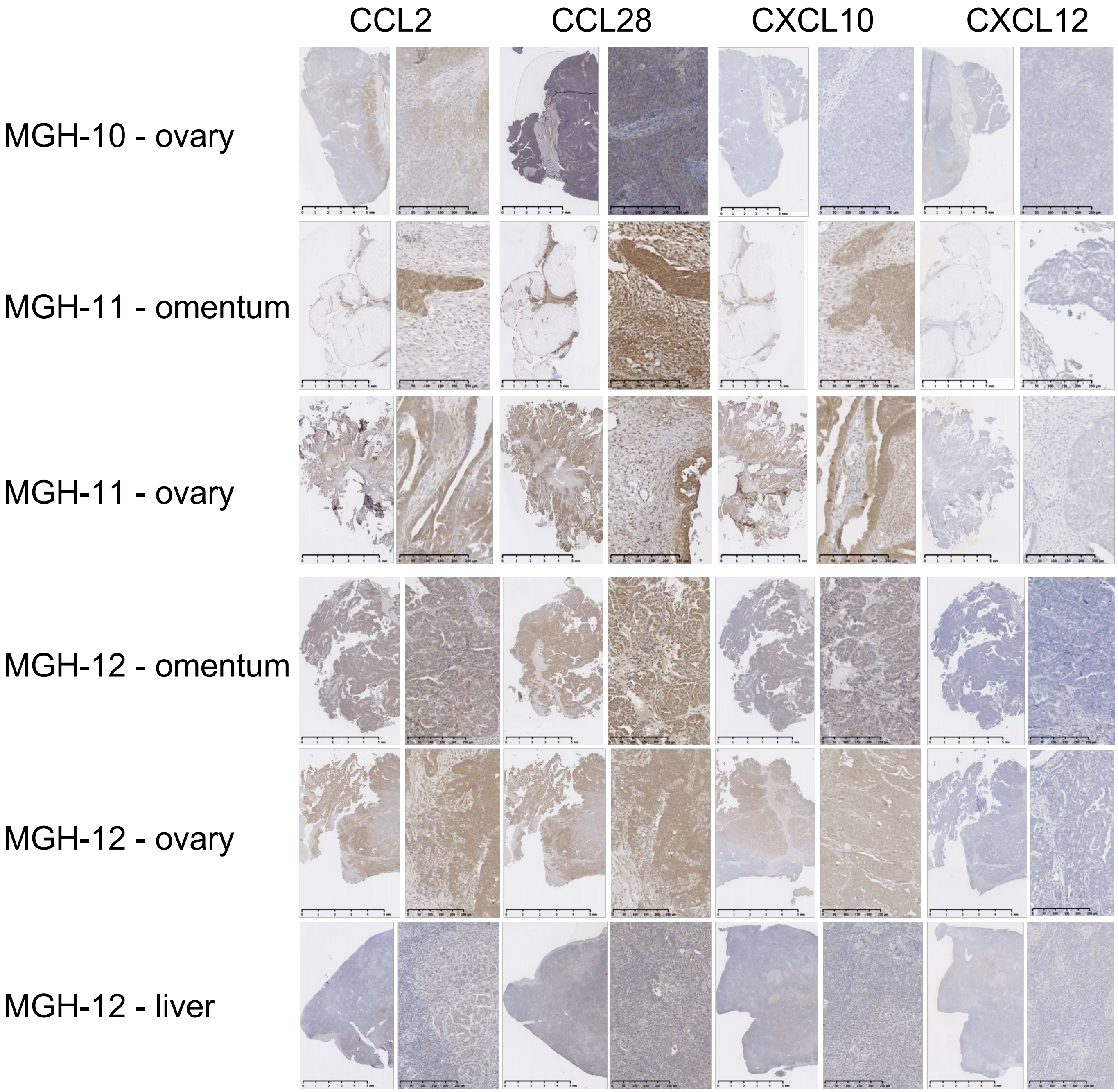
CCL2
CCL28
CXCL10
CXCL12
MGH-10 - ovary
MGH-11 - omentum
MGH-11 - ovary
MGH-12 - omentum
MGH-12 - ovary
MGH-12 - liver

Supplementary Figure 2

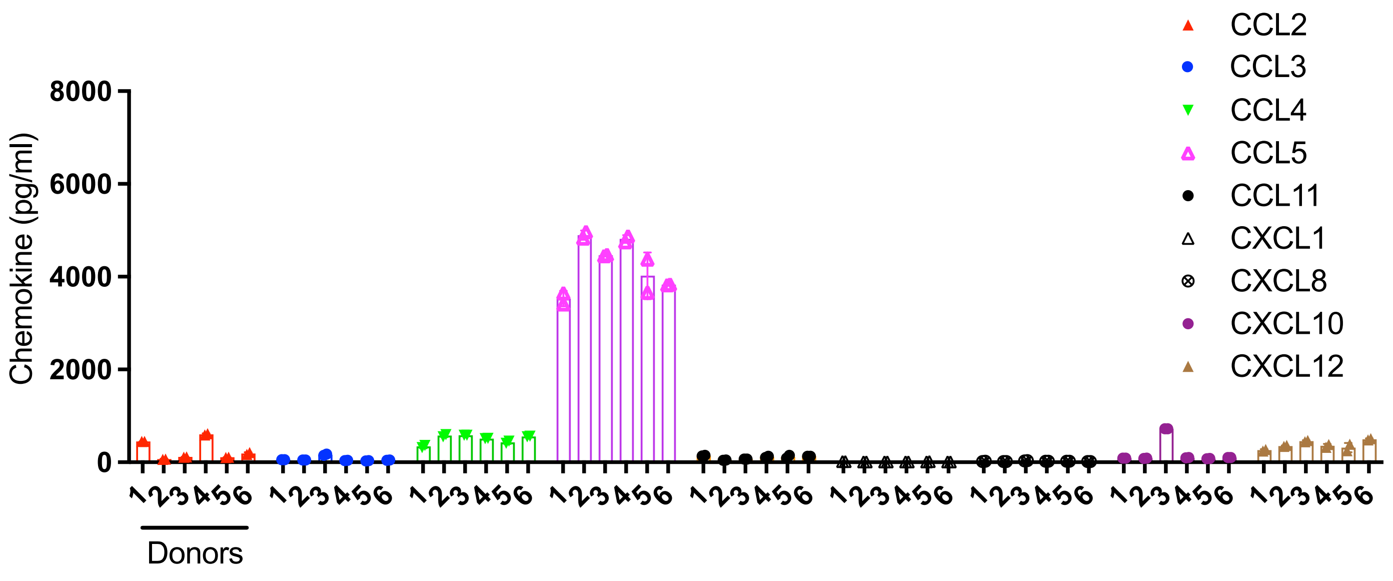

Supplementary Figure 3

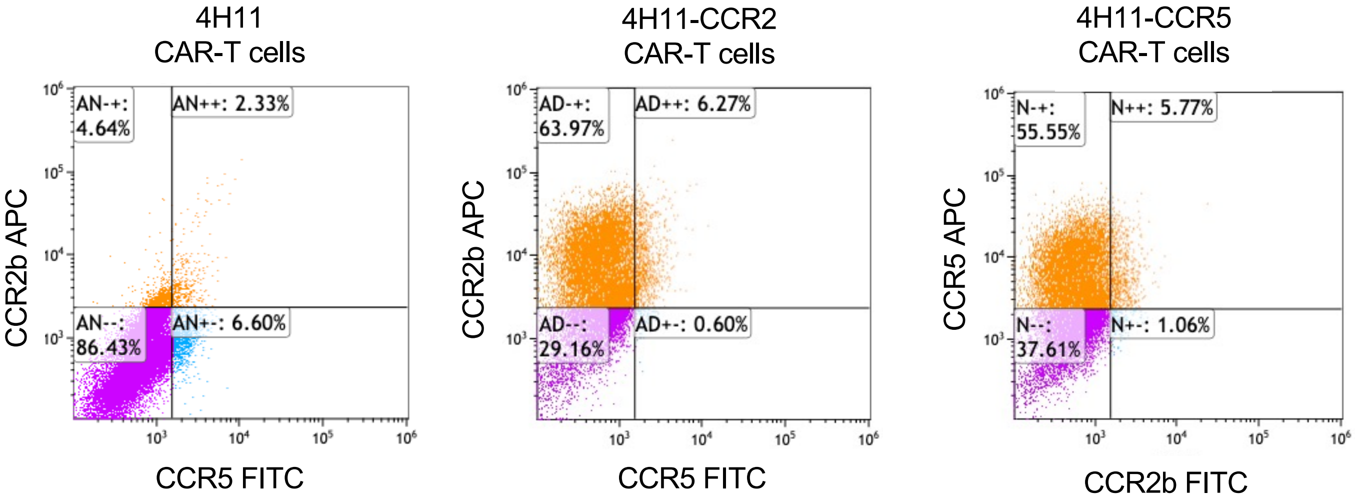

Supplementary Table 1

	CCL2	CCL-28	CXCL10	CXCL12
MGH-1 (Omentum)	2+	2+	1+	0
MGH-1 (Adnexa)	1+	0	1+	0
MGH-2 (Omentum)	0	2+	2+	0
MGH-2 (large bowel)	0	0	0	0
MGH-3 (Omentum)	1+	2+	0	1+
MGH-3 (large bowel)	1+	0	0	0
MGH-4 (Omentum)	2+	2+	0	2+
MGH-5 (Omentum)	2+	2+	2+	0
MGH-5 (L- Ovary)	1+	1+	0	0
MGH-6 (Omentum)	2+	2+	2+	0
MGH-6 (L- Ovary)	1+	2+	1+	0
MGH-7 (Omentum)	1+	2+	1+	0
MGH-8 (Omentum)	1+	2+	0	0
MGH-8 (R-Ovary)	0	1+	0	0
MGH-9 (Omentum)	1+	1+	0	1+
MGH-9 (Ovary)	2+	2+	0	0
MGH-9 (Liver)	2+	2+	2+	0
MGH-10 (Omentum)	1+	2+	1+	0
MGH-10 (Ovary)	1+	1+	0	0
MGH-11 (Omentum)	2+	2+	2+	0
MGH-11 (Ovary)	2+	2+	1+	0
MGH-12 (Omentum)	2+	2+	1+	0
MGH-12 (Ovary)	2+	2+	1+	0
MGH-12 (Liver)	1+	1+	0	1+
